# LncRNA-HNF1A-AS1 functions as a competing endogenous RNA to activate PI3K/AKT signalling pathway by sponging miR-30b-3p in gastric cancer

**DOI:** 10.1038/s41416-020-0836-4

**Published:** 2020-04-27

**Authors:** Hai-Ting Liu, Ran-Ran Ma, Bei-Bei Lv, Hui Zhang, Duan-Bo Shi, Xiang-Yu Guo, Guo-Hao Zhang, Peng Gao

**Affiliations:** 1 Key Laboratory for Experimental Teratology of the Ministry of Education and Department of Pathology, School of Basic Medical Sciences, Shandong University, Jinan, P. R. China; 2 Department of Pathology, Qilu Hospital, Shandong University, Jinan, P. R. China; 3 Department of Pathology, Shandong Provincial Hospital affiliated to Shandong university, Jinan, P. R. China

**Keywords:** Gastrointestinal cancer, Cell migration

## Abstract

**Background:**

Accumulating evidence demonstrated that long noncoding RNAs (lncRNAs) played important regulatory roles in many cancer types. However, the role of lncRNAs in gastric cancer (GC) progression remains unclear.

**Methods:**

RT-qPCR assay was performed to detect the expression of HNF1A-AS1 in gastric cancer tissues and the non-tumourous gastric mucosa. Overexpression and RNA interference approaches were used to investigate the effects of HNF1A-AS1 on GC cells. Insight into competitive endogenous RNA (ceRNA) mechanisms was gained via bioinformatics analysis, luciferase assays and an RNA-binding protein immunoprecipitation (RIP) assay, RNA-FISH co-localisation analysis combined with microRNA (miRNA)-pulldown assay.

**Results:**

This study displayed that revealed expression of HNF1A-AS1 was associated with positive lymph node metastasis in GC. Moreover, HNF1A-AS1 significantly promoted gastric cancer invasion, metastasis, angiogenesis and lymphangiogenesis in vitro and in vivo. In addition, HNF1A-AS1 was demonstrated to function as a ceRNA for miR-30b-3p. HNF1A-AS1 abolished the function of the miRNA-30b-3p and resulted in the derepression of its target, PIK3CD, which is a core oncogene involved in the progression of GC.

**Conclusion:**

This study demonstrated that HNF1A-AS1 worked as a ceRNA and promoted PI3K/AKT signalling pathway-mediated GC metastasis by sponging miR-30b-3p, offering novel insights of the metastasis mechanism in GC.

## Introduction

Gastric cancer (GC) is one of the most common carcinomas and is the fourth leading cause of cancer-related deaths globally.^1^ Prognoses are poor, and recurrence rates are high for the patients with GC due to tumour progression.^2^ Thus, developing effective targeted therapies for successful intervention is vitally important.^3^ Invasion and metastasis are essential processes for tumour and progression. However, the underlying molecular mechanisms that mediate invasion and metastasis have yet to be fully clarified.

Long noncoding RNAs (LncRNAs) are transcripts that lack protein-coding potential, and are at least 200 nucleotides in length.^4^ Recently, emerging evidence has implicated that lncRNAs played important roles in multiple biological functions, including proliferation, apoptosis, metastasis, angiogenesis and lymphangiogenesis.^5–7^ LncRNAs mediate multilevel regulation of gene expression, including transcriptional regulation by recruiting chromatin-modifying complexes and post-transcriptional regulation by directly binding to microRNAs, mRNAs or proteins.^8^ Some lncRNAs were identified as competing endogenous RNAs (ceRNAs) to sponge miRNAs, thus regulating cell biological functions.^9^ For example, LncRNA SNHG7, functioning as a ceRNA, enhances GALNT1-induced proliferation and metastasis via sponging miR-216b in colorectal cancer.^10^

Among all known cancer-related lncRNAs, HNF1A-AS1 originally was shown to be involved in human cancer development, and was identified as a potential biomarker.^11,12^ Yang et al. reported that HNF1A-AS1 was involved in oesophageal tumorigenesis by promoting cancer cell proliferation, invasion and metastasis via modulation of chromatin and nucleosome assembly as well as H19 induction.^12^ Fang et al. demonstrated that HNF1A-AS1 acted as an oncogene in colon cancer in part through serving as a ceRNA to modulate miRNA-34a expression, subsequently repress miR-34a/SIRT1/p53 feedback loop and then activate canonical Wnt signalling pathway.^13^

In our previous study, we found that EGR1-activated HNF1A-AS1 transcription promoted cell growth and cell cycles in GC, and that it could also increase GC cell migration abilities.^14^ At present, whether HNF1A-AS1 was dysregulated in GC needs to be addressed, as well as the association between HNF1A-AS1 expression and the clinicopathological parameters of the patients with GC. In addition, since the complexity of the internal and external environments in organisms is very complex and cannot be captured by in vitro models, whether HNF1A-AS1 can promote GC metastasis in vivo was investigated. Moreover, the underlying mechanism by which HNF1A-AS1 contributes to the GC metastasis is explored.

## Materials and methods

### Clinical samples

Sixty-seven samples of fresh GC tissue and six samples of non-tumourous gastric mucosa tissue were obtained from the Qilu Hospital, Shandong Provincial Hospital and the First Affiliated Hospital of WeiFang Medical University. All samples were stored in liquid nitrogen. None of the patients had undergone treatment prior to surgery. The clinicopathological characteristics for all of the patients are shown in Table 1. The study was approved by the Research Ethics Committee of Shandong University.

**Table 1:** The association between HNF1A-AS1 expression and clinicopathological parameters.

Variables	Low expression of HNF1A-AS1	High expression of HNF1A-AS1	*P*-value
Tumour size			*P* = 0.2308
<5 cm	9	14	
>=5 cm	24	20	
Age (years)			*P* = 0.7029
<62	14	16	
>=62	19	18	
Gender			*P* = 0.4158
Female	6	9	
Male	27	25	
Differentiation			*P* = 0.1384
Well differentiated	1	0	
Poorly	17	11	
Moderately	15	23	
Lymph node metastasis			*P* = 0.0262
Yes	23	31	
No	10	3	

### Cell culture and transfection

Human gastric cancer cell lines (MKN-45 and BGC-823) were commercially obtained from the Shanghai Cancer Institute. Human umbilical vein endothelial cells (HUVECs) were generously provided by Dr. Hu (Shandong University). All cell lines were routinely maintained in RPMI 1640 supplemented with 10% foetal bovine serum. Human lymphatic endothelial cells (HLECs), generously provided by Dr. Li (Shandong University), were cultured in MEM (ScienCell, San Diego, CA, USA) with 10% FBS. Mycoplasma PCR testing of these cells was performed every month. Cells were grown for no more than 15 passages in total for any experiment. HNF1A-AS1 siRNA (si-HNF1A-AS1), and siRNA-negative control (si-NC) were designed and synthesised by RiboBio (Guangzhou, China). The sequence of si-HNF1A-AS1 is CCCTCC ATCTAACATTCAA. The miRNA mimics mentioned in this study and the negative control (NC) were synthesised by GenePharma (Shanghai, China). MKN-45 and BGC-823 were seeded on 6- or 12-well plates and transfected 48 h later using X-tremeGENE transfection reagent (Roche Applied Science, Indianapolis, IN, USA) or Lipofectamine 2000 (Invitrogen, Carlsbad, CA, USA), according to the manufacturer’s instructions.

### RNA fluorescent in situ hybridisation (RNA-FISH)

The subcellular localisation of HNF1A-AS1 in gastric cancer was detected using a FISH kit (RiboBio Co., LTD, Guangzhou, China). Briefly, gastric cancer cells were fixed in 4% paraformaldehyde, permeabilised in PBS containing 0.5% Triton X-100 and then incubated with a Cy3-labelled LncRNA-HNF1A-AS1 probe according to the manufacturer’s protocols. The 18 s probe was used as a cytoplasmic reference, and the U6 probe was used as a nuclear reference. Cell nuclei were stained with 4′,6-diamidino-2-phenylindole (DAPI) for 5 min at room temperature. Fluorescence images were obtained using a fluorescence microscope (Olympus, Japan). A FAM-conjugated miR-30b-3p probe was designed and purchased from GenePharma. The samples were added with Cy3-labelled HNF1A-AS1 and FAM-conjugated miR-30b-3p probe. The co-localisation assay of HNF1A-AS1 and miR-30b-3p was observed by confocal microscopy (ZEISS, Germany).

### Real-time quantitative RT-PCR

Total RNA from gastric tissue samples and cancer cells was extracted using Trizol reagent (Invitrogen, Carlsbad, CA, USA) in accordance with the manufacturer’s protocol. Total RNA was reverse-transcribed to cDNA using a Reverse Transcription Kit (Toyobo Co., Ltd., Japan). Quantitative PCR was performed using SYBR Green (Roche Applied Science, Indianapolis, IN, USA). The expression level of each specific gene was normalised to that of GAPDH. The All-in-One^TM^ miRNA qRT-PCR Detection Kit (Genecopoeia, USA) was used for miRNA RT-qPCR. The expression level of miR-30b-3p was detected using miR-30b-3p-specific primer (Genecopoeia). U6 small nuclear RNA was used as the internal control. The relative gene expression was calculated using the 2^–ΔΔCt^ method.

### Plasmid construction

HNF1A-AS1 cDNA was subcloned into the pcDNA3.1 vector and named pcDNA3.1-HNF1A-AS1. Point mutations were introduced into the miR-30b-3p response elements in pcDNA3.1-HNF1A-AS1, producing a new plasmid designated as HNF1A-AS1-mut (miR-30b-3p). The HNF1A-AS1 cDNA was subcloned into the pmiRGLO vector and named pmiRGLO-HNF1A-AS1. The pmiRGLO-HNF1A-AS1 with point mutations in the miR-30b-3p response elements was named pmiRGLO-MUT-1. The 3′ untranslated regions (3′-UTR) of PIK3CD and AKT3, containing the intact miR-30b-3p recognition sequences, respectively, were subcloned into the pmirGLO vector and named pmiRGLO-PIK3CD or pmiRGLO-AKT3. The pmiRGLO-PIK3CD with point mutations in the miR-30b-3p response elements was named pmiRGLO-MUT (miR-30b-3p). The 3′-UTR of PIK3CD was subcloned into the pcDNA3.1 vector and named pcDNA3.1-PIK3CD 3′-UTR.

### Dual-luciferase reporter assay

MKN-45 and BGC-823 cells were co-transfected with wild- or mutant-type pmiRGLO plasmid and miRNA mimics using Lipofectamine 2000 (Invitrogen, Carlsbad, CA, USA). Forty-eight hours after transfection, the relative luciferase activity was measured using the dual-luciferase reporter system as previously reported.^15^

### Western blotting

The anti-PIK3CD antibody (1:500 dilution), anti-PIK3R1 antibody (1:1000 dilution), anti-AKT1 antibody (1:1000 dilution), anti-AKT2 antibody (1:300 dilution), anti-AKT3 antibody (1:500 dilution) and anti-β-actin antibody (1:1000 dilution) were incubated with polyvinylidene difluoride membranes at 4 °C overnight. β-actin was used as an endogenous control. A previously described detailed procedure is available.^16^

### Enzyme-linked immunosorbent assay (ELISA)

Cell culture supernatants of MKN-45 and BGC-823 cells that were transiently transfected with the pcDNA3.1-HNF1A-AS1 and pcDNA3.1 were collected and centrifuged at 3,000 rpm for 10 min. VEGF-C and VEGF-A secretion levels were detected via ELISA according to the manufacturer’s protocol (Multi Sciences Biotech, Co., Ltd., Hangzhou, China).

### Migration and invasion in vitro assays of GC cells

The migration and invasion abilities of GC cells were observed in both Matrigel assay-coated and uncoated Transwell chambers. A detailed procedure is described in a previous study.^17^

### Migration, proliferation and tube-formation assay in HUVECs and HLECs

Migration, proliferation, and tube-formation assay in HUVECs and HLECs were performed as described in previous study.^17,18^

### RNA-binding protein immunoprecipitation (RIP) assay

RNA immunoprecipitation was performed using the EZ-Magna RIP RNA-binding protein immunoprecipitation kit (Millipore, Billerica, MA, USA) and the Argonaute2 (Ago2, Millipore) antibody in accordance with the manufacturer’s protocol. The cells were lysed using RIP lysis buffer. A 100-μL volume of whole-cell lysate was incubated with RIP buffer containing magnetic beads conjugated with human anti-Ago2 antibody (Millipore) and normal mouse immunoglobulin G (IgG, Millipore), which served as a negative control. Samples were incubated with Proteinase K buffer and immunoprecipitated RNA was then extracted. RT-qPCR analysis, using Fos and HNF1A-AS1 primers, was performed to identify the presence of binding targets in the co-precipitated RNAs.

### Biotin-labelled pull-down assays

MKN-45 and BGC-823 cells were transfected with biotinylated miR-30b-3p and a scrambled control (GenePharma, Shanghai) and collected 48 h after transfection. The cell lysates were incubated with M-280 streptavidin magnetic beads (Invitrogen, San Diego, CA, USA) and 10 μl of yeast tRNA on a rotator at 4 °C for 2 h. Add 750 μl of Trizol (Invitrogen, Carlsbad, CA) and 250 μl of water to the input, and the pull-down beads and the bound RNAs were purified. The 3′ biotin-labelled miR-30b-3p sequence is 5′-CUGGGAGGUGGAUGUUUACUUC-3′ BiO. The scrambled control miRNA sequence is 5′-UUCUCCGAACGUGUCACGUTT-3′ BiO.

### Mouse tumour xenograft experiments

Four-week-old male Nu/nu athymic nude mice were obtained from the National Laboratory Animal Center (Weitonglihua Biotechnology, Beijing, China) and were maintained under SPF room conditions for feeding and observation. The mice were randomly divided into four groups. MKN-45 cells were transfected with lentivirus vector LV5-GFP-HNF1A-AS1 or LV5-GFP-Negative control (GenePharma Co., Ltd., Shanghai, China). The titre of lentivirus was 1 × 10^9^ TU/ml. HNF1A-AS1 was initiated by the EF-1a promoter, with the full sequence map for LV5 shown in Supplementary Fig. 1a. A total of 2 × 10^6^ LV5-HNF1A-AS1 or LV5-NC cells were then injected into the lateral tail vein or the axillary fossa of each mouse (*n* = 6 mice per group) on 18 September 2017. To ameliorate pain to the mice throughout experimental studies, nasal anaesthesia (isoflurane) was introduced. The benefits of isoflurane anaesthesia for animals are that the anaesthesia depth was easy to control, and isoflurane did not affect body metabolism. Mice were anaesthetised using an anaesthetic machine (Kodak, Rochester, USA) with MAC 1.6% isoflurane. All experiments were performed inside a biosafety cabinet during the animal’s light time cycle on the first floor of the Experimental Animal Room at Shandong University. The mice were killed by cervical dislocation, and tumour, lung, liver and kidney were isolated from the mice for further analysis. All of the animal experiments were conducted according to the Guidelines for Animal Health and Use (Ministry of Science and Technology, China, 2006). Animal experiments were approved by the Committee for Animal Protection and Utilisation of Shandong University.

### Immunohistochemistry for CD-34

Immunohistochemical (IHC) analysis for CD-34 was performed to determine the amount of blood isolated from the mouse xenograft tumour tissues. Procedures were performed, and the results assessed as previously reported.^19^

### Statistical analysis

Statistical analyses were performed using GraphPad Prism Software (GraphPad Software, Inc., La Jolla, CA, USA). Significant differences between the two groups were assessed via the Student’s *t* test. The χ^2^ test was used to analyse the association between HNF1A-AS1 expression and clinicopathological parameters. Statistical significance was considered as *P* < 0.05.

## Results

### High expression of HNF1A-AS1 is correlated with lymph node metastasis in GC patients

**Fig. 1:**
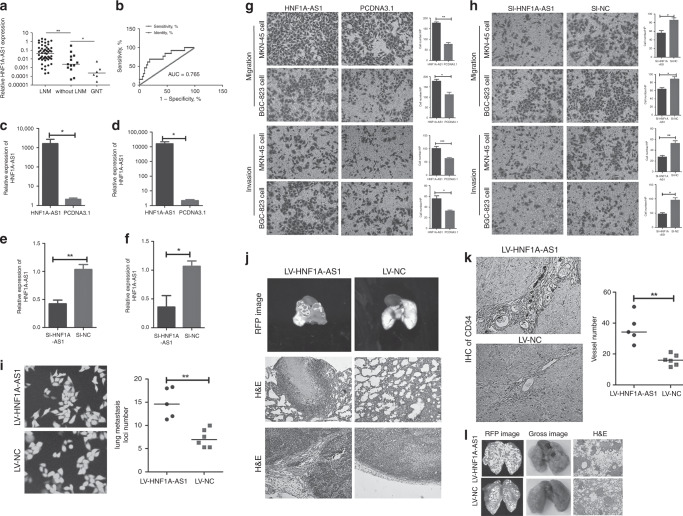
HNF1A-AS1 promotes the migration, invasion and metastasis abilities of GC cells in vivo and in vitro. **a** HNF1A-AS1 expression was demonstrated by RT-qPCR assays in human gastric cancer tissues with lymph node metastasis (LNM), without lymph node metastasis (without LNM) and non-tumour gastric mucosa. **b** Receiver- operating characteristic (ROC) curve showed that HNF1A-AS1 expression could effectively distinguish the cases with LNM from those without LNM in patients with gastric cancers. **c**–**f** The overexpression (**c**, **d**) and knockdown (**e**, **f**) efficiency of HNF1A-AS1 in both GC cells. **g** Transwell assays demonstrated that HNF1A-AS1 enhanced cell migration and invasion abilities in MKN-45 and BGC-823 cells (×200). Three independent experiments were performed, and data are presented as mean ± SD. **h** Transwell assays indicated that HNF1A-AS1 knockdown inhibited cell migration and invasion abilities in MKN-45 and BGC-823 cells (×200). Three independent experiments were performed, and data are presented as mean ± SD. **i** HNF1A-AS1 was overexpressed stably in MKN-45 cells, with GFP as marker gene (left, ×200). The number of lung metastatic foci in the LV5-HNF1A-AS1 group was more than that in the LV5-NC group (right). **j** In total, 1.5 × 10^6^ MKN-45 cells transfected with LV5-HNF1A-AS1 vector or LV5-NC vector were injected into the axillary fossa of the mice. Compared with the LV5-NC group, more stroma, muscle invasion or lung metastasis was observed in the LV5-HNF1A-AS1 group. Representative lung metastasis locus was shown in IVIS system imaging (top) and H&E staining (middle, ×100). Representative stromal invasion was shown in H&E staining (bottom, ×100). **k** IHC assays of CD-34 showed that the blood vessel density in LV5-HNF1A-AS1 group remarkably increased than that in the LV-NC group (×100). **l** In total, 2.5 × 10^6^ MKN-45 cells transfected with LV5-HNF1A-AS1 vector or LV5-NC vector were injected into the tail vein of the mice. The number of lung metastatic foci in the LV5-HNF1A-AS1 group was more than that in the LV5-NC group. Representative lung metastasis loci were shown in IVIS system imaging (left), bright-field gross imaging (middle, ×100) and H&E staining (right, ×100), with the quantifiable results shown in Fig. 1i (right). *P < 0.05, **P < 0.01, ***P < 0.001.

HNF1A-AS1 expression was measured in human GC tissues and non-tumourous gastric mucosa tissues. HNF1A-AS1 in gastric cancer has been found to be significantly overexpressed compared with human non-tumourous gastric mucosa tissue. Further upregulation of HNF1A-AS1 was observed in GC cases with lymph node metastasis (LNM) when compared with GC cases without LNM (Fig. 1a). The expression of HNF1A-AS1 was classified as high or low expression based on a median score. The clinicopathological assay revealed that higher HNF1A-AS1 expression in GC tissues was remarkably correlated with positive LNM (*P* = 0.0262), but there was no correlation between HNF1A-AS1 expression and tumour size (*P* = 0.2308), age (*P* = 0.7029), gender (*P* = 0.4158) or differentiation (*P* = 0.1384) (Table 1). A receiver-operating characteristic (ROC) curve assay was performed to evaluate whether HNF1A-AS1 expression could be used to distinguish between patients with LNM and those without LNM. The AUC value for HNF1A-AS1, distinguishing GC cases with LNM from those without LNM, was as high as 0.7650 (Fig. 1b, 95% confidence interval (CI) = 0.6177–0.9122, *P* = 0.0032). These results imply that HNF1A-AS1 expression is associated with LNM and may serve as a biomarker for predicting GC LNM.

### HNF1A-AS1 promotes cell migration, invasion and metastasis in GC

To further explore the biological functions of HNF1A-AS1 in GC cells, GC cells were transfected with the pcDNA3.1-HNF1A-AS1 plasmid or siRNAs against HNF1A-AS1. The overexpression and knockdown efficiency of HNF1A-AS1 was detected 48 h after transfection (Fig. 1c, d, e, f). HNF1A-AS1 significantly promoted cell migration and invasion, while HNF1A-AS1 knockdown markedly suppressed cell migration and invasion (Fig. 1g, h). MKN-45 cells transduced with LV5-HNF1A-AS1/LV5-NC expressing green fluorescent protein/GFP (Fig. 1i) were injected into the axillary fossa of mice to investigate the functional roles of HNF1A-AS1 in vivo. RT-qPCR assay showed that HNF1A-AS1 was stably overexpressed in MKN-45 cells in vivo (Supplementary Fig. 1b). Local invasion occurred in all of the mice with LV5-HNF1A-AS1-transfected tumours. In contrast, only two of six LV5-NC mice developed local invasion; the other four mice had tumours that were well-encapsulated with non-invasive margins (Fig. 1j). Moreover, we observed that the number of CD-34-positive cells was higher in the LV5-HNF1A-AS1 group compared with the control group by IHC analysis (Fig. 1k), indicating that HNF1A-AS1 promoted angiogenesis in GC. In addition, metastatic cancer cells were observed in the lungs of mice in the HNF1A-AS1 group, which may be due to HNF1A-AS1-mediated angiogenesis in GC (Fig. 1j). In homogeneous metastatic models, mice in the LV5-HNF1A-AS1 group had a greater number of more metastatic loci in the lungs compared with mice in the LV5-NC group (14.6 ± 3.24 loci vs. 6.9 ± 1.9 loci, respectively, Fig. 1i, l). Taken together, these results suggest that HNF1A-AS1 increased the invasion, metastasis and angiogenesis of GC in vivo.

### HNF1A-AS1 promotes angiogenesis of HUVECs and lymphangiogenesis of HLECs

**Fig. 2:**
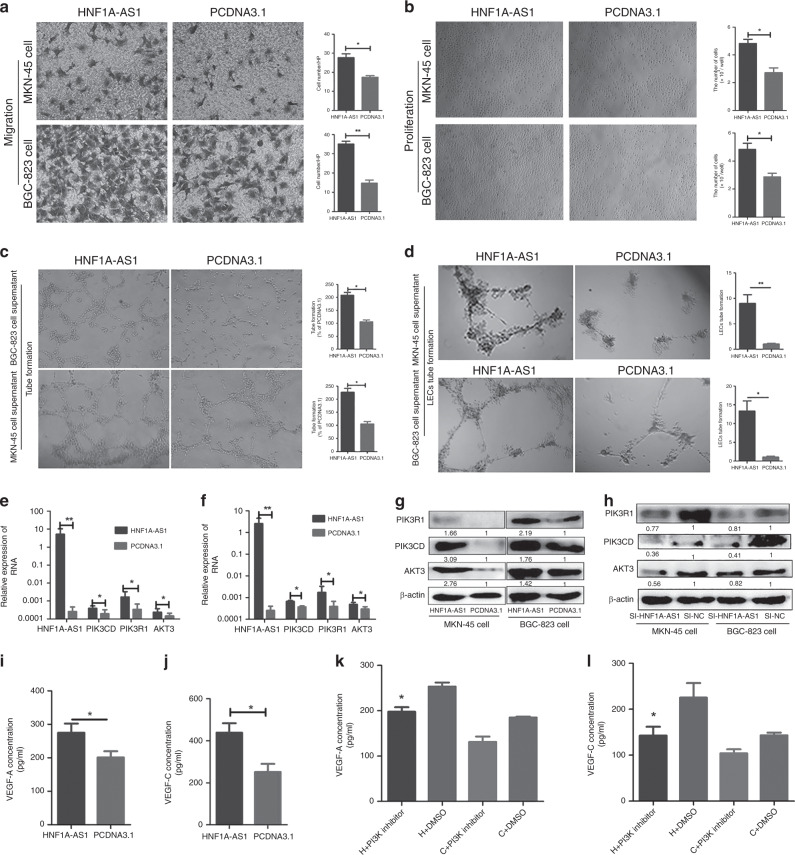
HNF1A-AS1 promotes HUVEC angiogenesis, HLEC lymphangiogenesis and regulates PIK3CD, PIK3R1 and AKT3 expression. **a**–**d** Cell culture supernatants of MKN-45 and BGC-823 cells transiently transfected with the HNF1A-AS1 or the negative control were collected. The cell culture supernatant from the HNF1A-AS1-transfected groups rather than the negative control groups promoted the migration (**a**, ×200), proliferation (**b**, ×40) and tube formation (**c**, ×40) capabilities of HUVECs and also enhanced tube- formation abilities of HLECs (**d**, ×40). Three independent experiments were performed, and data are presented as mean ± SD. **e**, **f** RT-qPCR assays showed that HNF1A-AS1 overexpression increased PIK3CD, PIK3R1 and AKT3 mRNA level in MKN-45 cells (**e**) and BGC-823 cells (**f**). Three independent experiments were performed, and data are presented as mean ± SD. **g** Western blotting assays demonstrated that HNF1A-AS1 overexpression enhanced PIK3CD, PIK3R1 and AKT3 protein expression. Three independent experiments were performed, and data are presented as mean ± SD. **h** Western blotting assays demonstrated that knockdown of HNF1A-AS1 by siRNA decreased PIK3CD, PIK3R1 and AKT3 protein expression. Three independent experiments were performed, and data are presented as mean ± SD. **I**, **j** The secretion level of VEGF-A (**i**) and VEGF-C (**j**) significantly enhanced in the culture supernatant from the HNF1A-AS1-transfected MKN-45 cells, compared with the negative control groups, detected by ELISA. Three independent experiments were performed, and data are presented as mean ± SD. **k**, **l** The GC cell line MKN-45 cells were transfected with pcDNA3.1 or pcDNA3.1-HNF1A-AS1, and the PI3K/AKT inhibitor NVP-BKM-120 (1 μM) was then added; DMSO was added as a control. In pcDNA3.1-HNF1A-AS1-transfected GC cells, VEGF-A (**k**) and VEGF-C (**l**) protein levels decreased significantly with NVP-BKM-120 treatment compared with those of DMSO-treated GC cells. *P < 0.05, **P < 0.01, ***P < 0.001.

Next, we explored the role of HNF1A-AS1 in GC angiogenesis and lymphangiogenesis in vitro. The Transwell migration assay revealed that the migration activity of HUVECs dramatically increased when cultured with conditioned medium from MKN-45 and BGC-823 cells transfected with pcDNA3.1-HNF1A-AS1 (Fig. 2a). In addition, HUVEC proliferation was significantly enhanced in the HNF1A-AS1 overexpression group (Fig. 2b). Matrigel-based capillary tube-formation assays were subsequently performed to evaluate the role of HUVECs in tube formation. Tube-formation ability was increased in the HNF1A-AS1-overexpression group compared with the control group (Fig. 2c). In addition, our results showed that HNF1A-AS1 promoted HLEC tube formation compared with the control group (Fig. 2d). The above observations indicate that HNF1A-AS1 overexpression stimulates GC angiogenesis and lymphangiogenesis in vitro.

### HNF1A-AS1 regulates PIK3CD, PIK3R1 and AKT3 expression

**Fig. 3:**
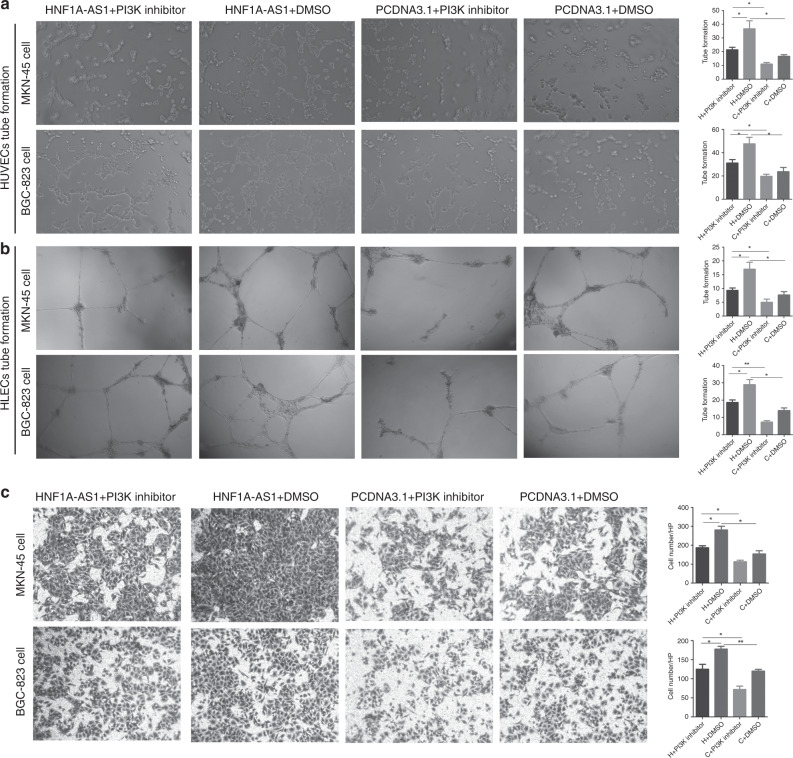
The effect of PI3K inhibition on HNF1A-AS1-mediated biological functions. **a**–**c** The GC cell line MKN-45 cells were transfected with pcDNA3.1 or pcDNA3.1-HNF1A-AS1, and the PI3K/AKT inhibitor NVP-BKM-120 was then added; DMSO was added as a control. NVP-BKM120 (PI3K inhibitor) treatment rescued the tube formation of HUVECs (**a**, ×40) and HLECs (**b**, ×40), and migration abilities (**c**, ×100) of GCs enhanced by HNF1A-AS1. *P < 0.05, **P < 0.01, ***P < 0.001.

To better examine the detailed regulatory mechanism of HNF1A-AS1 in GC metastasis, the PI3K/AKT signalling pathway that was involved in cancer cell invasion,^20^ angiogenesis^21^ and lymphangiogenesis^22^ was studied. PI3K/AKT signalling pathway was highly expressed and promoted GC progression.^23,24^ Furthermore, PIK3CD, PIK3R1, and AKT3, rather than AKT1 and AKT2, were observed to be notably upregulated in the HNF1A-AS1 overexpression group (Fig. 2e, f, g; Supplementary Fig. 1c, d, e), and were downregulated in the HNF1A-AS1-knockdown group at both the mRNA and protein levels (Fig. 2h; Supplementary Fig. 1f, g, h, i, j). Recent studies showed that the activation of the PI3K/AKT pathway in tumour cells increases the secretion of VEGF-A^25^ and VEGF-C.^26^ VEGF-A exerts a pro-angiogenic role in human cancer,^27^ and VECF-C is a well-known regulator of the lymphangiogenesis.^28^ Therefore, an ELISA assay was performed to demonstrate the effect of HNF1A-AS1 on VEGF-A and VEGF-C secretion. Interestingly, the VEGF-A and VEGF-C secretion levels in the supernatants isolated from MKN-45 and BGC-823 cells transfected with the HNF1A-AS1-overexpression vector were respectively significantly improved compared with control cells (Fig. 2i, j; Supplementary Fig. 1k), indicating that PI3K/AKT signalling pathway may be involved in HNF1A-AS1-induced VEGF-A and VEGF-C secretion. Next, we demonstrated the effect of a PI3K inhibitor (NVP-BKM120) on HNF1A-AS1-mediated VEGF-A and VEGF-C secretion. Our results showed that the secretion levels of VEGF-A and VEGF-C were decreased in LV5-HNF1A-AS1 GCs with NVP-BKM120 treatment (1 μM) compared with the control cells treated only with DMSO (Fig. 2k, l). A rescue experiment was also performed to confirm that HNF1A-AS1 exerts its biological functions through PI3K/AKT signalling. Interestingly, NVP-BKM120 treatment rescued the tube formation of HUVECs and HLECs, and the migration abilities of GCs enhanced by HNF1A-AS1 (Fig. 3a, b, c). Taken together, these results indicated that the HNF1A-AS1-induced biological functions might be mediated by PI3K/AKT signalling pathway.

### HNF1A-AS1 functions as a ceRNA with miR-30b-3p

**Fig. 4:**
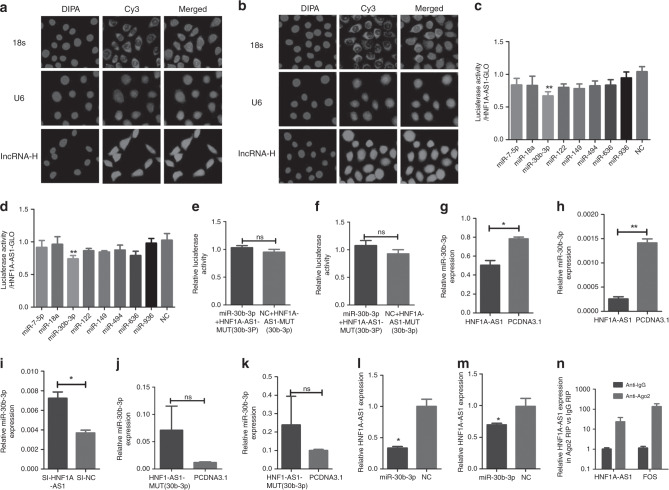
HNF1A-AS1 functions as a ceRNA with miR-30b-3p. **a**, **b** RNA fluorescent in situ hybridisation of the LncRNA-HNF1A-AS1 in MKN-45 cells (**a**) and BGC-823 cells (**b**). The 18 s and U6 probe were respectively used as a cytoplasm and nucleus control (×200). **c**, **d** Luciferase assays showed that miR-30b-3p decreased luciferase activity of pmirGLO-HNF1A-AS1 in MKN-45 (**c**) and BGC-823 (d) cells. **e**, **f** Luciferase assays indicated that miR-30b-3p did not decrease luciferase activity of MUT-1 in MKN-45 cells (**e**) and BGC-823 cells (**f**). **g–k** The expression level of miR-30b-3p was detected by RT-qPCR following HNF1A-AS1 overexpression (**g**, **h**), knockdown (**i**) and HNF1A-AS1-MUT-30b-3p (**j**, **k**) overexpression in GC cells. **l**, **m** RT-qPCR assays showed that miR-30b-3p decreases the expression level of HNF1A-AS1 in MKN-45 cells (**l**) and BGC-823 cells (**m**). **n** HNF1A-AS1 was enriched in Ago2 immunoprecipitates relative to control IgG immunoprecipitates from BGC-823 cell extracts. All the above experiments were performed in three independent experiments, and data are presented as mean ± SD. *P < 0.05, **P < 0.01, ***P < 0.001.

Recently, accumulating evidence has demonstrated that lncRNAs can function as ceRNAs to sponge miRNAs, resulting in decreased miRNA expression in the cytoplasm and modulating the derepression of miRNA targets at the post-transcriptional level.^29^ HNF1A-AS1 was found to be distributed in both the nucleus and cytoplasm of gastric cancer cells via RNA-FISH (Fig. 4a, b). To evaluate whether HNF1A-AS1 functions as ceRNA, miRNAs were predicted to be bound to HNF1A-AS1 and PIK3CD, PIK3R1 or AKT3, using programmes, such as RegRNA, miRBase, miRwalk and targetscan. Based on these data and the previous reports about the candidate miRNAs’ function and the number of predicted target sites, we chose eight cancer-related or tumour-suppressing miRNAs for further investigation, including miR-7-5p,^30^ miR-18a,^31^ miR-30b-3p,^32^ miR-122,^33^ miR-149,^34^ miR-494,^35^ miR-636^36^ and miR-936.^37^ MiR-30b-3p decreased luciferase activity in pmirGLO-HNF1A-AS1 (Fig. 4c, d), but not in MUT-1 (Fig. 4e, f), indicating that the miRNA can directly bind to HNF1A-AS1 through their respective miRNA- binding sites (Supplementary Fig. 1l). In addition, HNF1A-AS1 overexpression decreased miR-30b-3p expression (Fig. 4g, h), while HNF1A-AS1 knockdown markedly enhanced miR-30b-3p expression (Fig. 4i). Overexpression of HNF1A-AS1-Mut (miR-30b-3p) did not decrease the expression of miR-30b-3p (Fig. 4j, k).

**Fig. 5:**
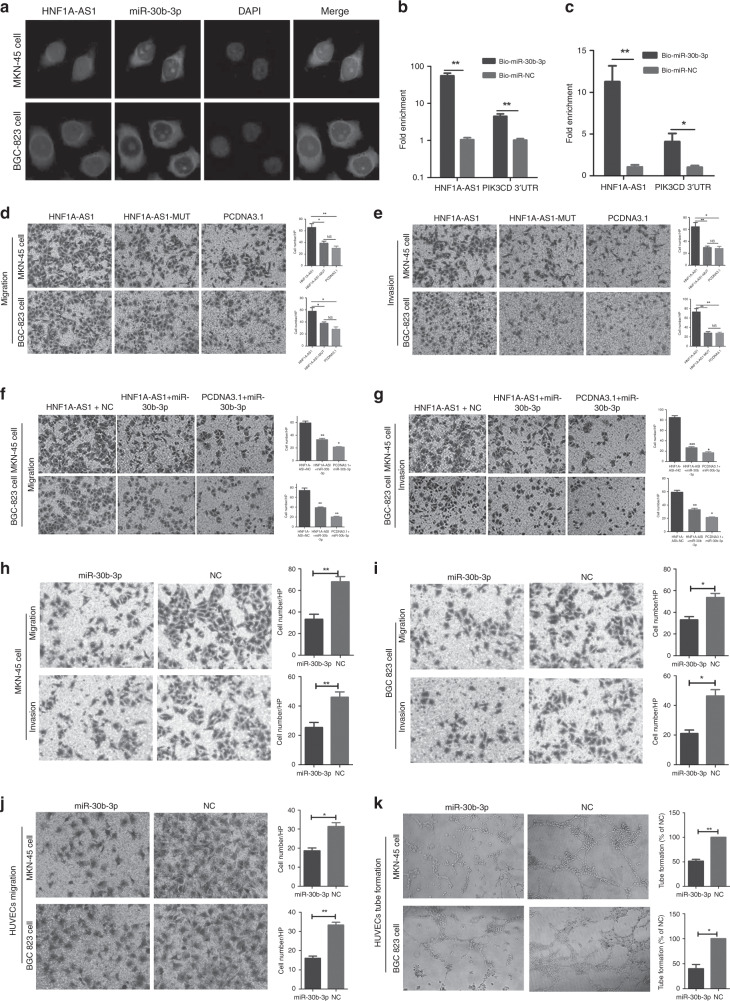
MiR-30b-3p abolished the HNF1A-AS1-mediated biological effects. **a** Co-localisation between HNF1A-AS1 and miR-30b-3p was observed by RNA-FISH in MKN-45 and BGC-823 cells; FAM-conjugated miR-30b-3p probes and Cy3-labelled HNF1A-AS1 probes were used for staining, followed by counterstain with DAPI (×200). **b**, **c** RT-qPCR analysis of HNF1A-AS1 or PIK3CD 3′-UTR levels in the streptavidin-captured fractions from MKN-45 cells (**b**) and BGC-823 cell (**c**) lysates after transfection with biotinylated miR-30b-3p or control NC. **d**, **e** Transwell assays demonstrated that HNF1A-AS1 with miR-30b-3p-mutated binding sites exerted a weaker effect on GC migration and invasion abilities compared with wild-type HNF1A-AS1 in MKN-45 cells (**d**) and BGC-823 cells (**e**) (×100). Three independent experiments were performed, and data are presented as mean ± SD. **f**, **g** The migration (**f**) and invasion (**g**) abilities were enhanced in HNF1A-AS1-overexpression group, while miR-30b-3p mimics reversed the promotion of migration and invasion capabilities by HNF1A-AS1 (×200). Three independent experiments were performed, and data are presented as mean ± SD. **h**, **i** MiR-30b-3p significantly inhibited migration and invasion abilities in MKN-45 (**h**) and BGC-823 cells (**i**) (×100). **j** Migration abilities of HUVECs were dramatically inhibited in both GC cells and transfected groups (×200). **k** MiR-30b-3p significantly inhibited tube-formation abilities of HUVECs in both GC cells and transfected groups (×40). All the above experiments were performed in three independent experiments, and data are presented as mean ± SD. *P < 0.05, **P < 0.01, ***P < 0.001.

Interestingly, miR-30b-3p notably decreased HNF1A-AS1 expression (Fig. 4l, m). It has been proposed that miRNAs directly regulate their targets and induce RNA degradation and/or translational repression through the formation of RNA-induced silencing complexes (RISCs) containing the Ago2 protein, a key component of RISC.^38^ To investigate whether HNF1A-AS1 interacts with RISC, RIP experiments were performed with BGC-823 cell extracts using antibodies against Ago2. The endogenous HNF1A-AS1 was specifically enriched in Ago2 immunoprecipitate compared with control, Immunoglobulin G (IgG) (Fig. 4n). Thus, HNF1A-AS1 was present in the RISCs containing Ago2, which is consistent with the data from the bioinformatics software analysis, as well as the luciferase and qRT-PCR assays. RNA-FISH assays showed that HNF1A-AS1 and miR-30b-3p were co-localised in the cytoplasm (Fig. 5a). In addition, a Biotin-labelled pull-down assay was applied to detect whether miR-30b-3p could directly interact with HNF1A-AS1. As shown in Fig. 5b, c, the level of HNF1A-AS1 was markedly elevated in the biotin-labelled miR-30b-3p-captured fraction compared with the negative control, suggesting that miR-30b-3p interacts with HNF1A-AS1 directly and in a sequence-specific manner.

Next, we explored the effects of HNF1A-AS1 with miR-30b-3p-mutated binding sites on GC cell migration and invasion abilities. Our data showed that HNF1A-AS1 with miR-30b-3p-mutated binding sites exerted only a weaker effect on GC migration and invasion abilities compared with the wild-type HNF1A-AS1 (Fig. 5d, e). In addition, a rescue experiment was then performed to explore whether HNF1A-AS1 exerts biological functions through miRNA. The migration and invasion abilities were enhanced in the HNF1A-AS1-overexpression group; however, the increased migration and invasion activities promoted by HNF1A-AS1 were reversed by miR-30b-3p mimics (Fig. 5f, g), suggesting that HNF1A-AS1 exerts its tumour-oncogene effects by repressing miRNAs in GC cells. Taken together, the above results indicate that HNF1A-AS1 acts as a ceRNA with miR-30b-3p.

### MiR-30b-3p exerts tumour-suppressive functions through regulation of PI3K/AKT signalling

To ascertain the biological functions of miR-30b-3p in GC cells, miR-30b-3p mimics were transfected into MKN-45 and BGC-823 cells (Supplementary Fig. 1m, n). MiR-30b-3p inhibited GC cell migration and invasion activities (Fig. 5h, i). The potential fundamental roles of miR-30b-3p in angiogenesis were then evaluated. The migration (Fig. 5j) and tube-formation abilities (Fig. 5k) of HUVECs were inhibited when cultured with conditioned medium from MKN-45 and BGC-823 cells transfected with miR-30b-3p mimics.

**Fig. 6:**
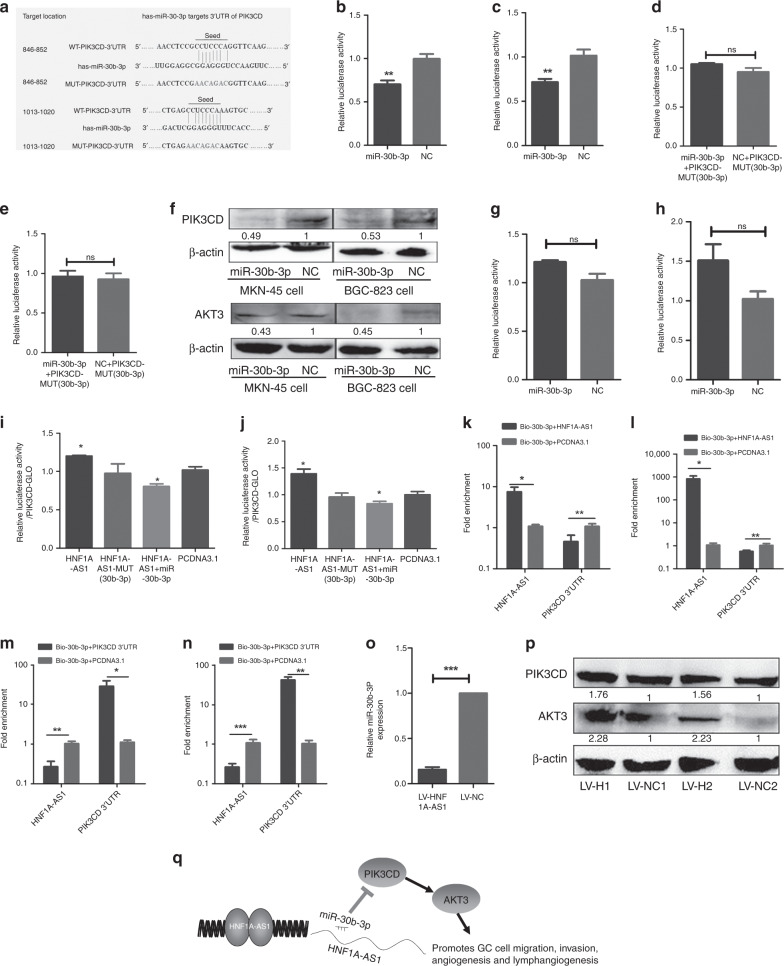
MiR-30b-3p exerts tumour-suppressive functions through regulation of PI3K/AKT signalling in GC. **a** The schematic of miR-30b-3p in PIK3CD 3′-UTR or mutant sequences. **b**–**e** MiR-30b-3p could reduce the luciferase activities in MKN-45 cells (**b**) and BGC-823 cells (**c**) transfected with the PIK3CD 3′-UTR-WT vector, while it had no effect on the PIK3CD 3′-UTR-MUT vector MKN-45 cells (**d**) and BGC-823 cells (**e**). **f** MiR-30b-3p suppressed the PIK3CD and AKT3 protein expression in MKN-45 and BGC-823 cells. **g**, **h** Luciferase activity assay showed that miR-30b-3p did not induce a significant decrease in luciferase activity of pmirGLO-AKT3, indicating that AKT3 is not a direct target gene of miR-30b-3p in MKN-45 (**g**) and BGC-823 cells (**h**). **I**, **j** Luciferase activity assay indicated that overexpression of HNF1A-AS1, but not the HNF1A-AS1 (miR-30b-mut), increased the luciferase activity of pmirGLO-PIK3CD, while ectopic expression of miR-30b-3p diminished this upregulation in MKN-45 (**i**) and BGC-823 cells (**j**). **k**, **l** RT-qPCR analysis of HNF1A-AS1 or PIK3CD 3′-UTR levels in the streptavidin-captured fractions from MKN-45 cells (**k**) and BGC-823 cell (**l**) lysates after transfection with biotinylated miR-30b-3p and pcDNA3.1-HNF1A-AS1 or control group. **m**, **n** RT-qPCR analysis of HNF1A-AS1 or PIK3CD 3′-UTR levels in the streptavidin-captured fractions from MKN-45 cells (**m**) and BGC-823 cell (**n**) lysates after transfection with biotinylated miR-30b-3p and pcDNA3.1-PIK3CD 3′-UTR or control group. **o**, **p** The expression of miR-30b-3p (**o**) was decreased, while the protein expression of PIK3CD and AKT3 (**p**) was increased in xenograft tumour tissues of the LV5-HNF1A-AS1 group, compared with those of the LV-NC group. **q** Graphic model as discussed in the text. lncRNA-HNF1A-AS1 acts as a ceRNA, activates PI3K/AKT signalling by competitively binding to miR-30b-3p, which exhibits oncogenic properties in GC. All the above experiments were performed in three independent experiments, and data are presented as mean ± SD. *P < 0.05, **P < 0.01, ***P < 0.001.

MiR-30b-3p was then investigated to determine whether it targeted PIK3CD, PIK3R1 and AKT3. Bioinformatic programmes (miRwalk and targetscan) revealed binding sites for miR-30-3p in the 3′-UTR of PIK3CD and AKT3, but not PIK3R1 (Fig. 6a). Luciferase assays demonstrated that miR-30b-3p suppressed the luciferase activity of the pmirGLO-PIK3CD-WT (Fig. 6b, c), but not pmirGLO-PIK3CD-Mut (miR-30b-3p) (Fig. 6d, e). Subsequently, miR-30b-3p significantly reduced PIK3CD protein levels (Fig. 6f), suggesting that PIK3CD is a direct target gene of the miRNA. A biotin-labelled pull-down assay was performed to detect whether miR-30b-3p could directly interact with PIK3CD. Our result showed that the level of PIK3CD 3′-UTR was markedly elevated in the biotin-labelled miR-30b-3p-captured fraction compared with that in the negative control (Fig. 5b, c), suggesting that miR-30b-3p interacted with PIK3CD 3′-UTR in a sequence-specific manner. In addition, neither miRNA induced a significant decrease in the luciferase activity of pmirGLO-AKT3 (Fig. 6g, h), indicating that AKT3 is not a direct target gene of miR-30b-3p. However, miR-30b-3p did decrease the AKT3 protein levels (Fig. 6f). AKT3 has previously been reported to function as a downstream gene in the PI3K signalling pathway, indicating that miR-30b-3p may indirectly reduce the AKT3 protein level, due to a decrease in PIK3CD.

As demonstrated, HNF1A-AS1 enhanced PIK3CD transcripts and protein levels. In order to examine whether HNF1A-AS1-mediated upregulation of PIK3CD relied on miRNAs, the miR-30b-3p-binding site was mutated, and luciferase activity assays were performed. As expected, overexpression of HNF1A-AS1, but not HNF1A-AS1 (miR-30b-3p-mut), increased the luciferase activity of pmirGLO-PIK3CD. Ectopic expression of miR-30b-3p diminished this upregulation (Fig. 6i, j), suggesting an essential role for HNF1A-AS1 in regulating PIK3CD by competitively binding to miRNAs. MiRNA pull-down assays were further performed to detect the competitive relationship between HNF1A-AS1 and PIK3CD. Our data indicated that the level of PIK3CD 3′-UTR was reduced in the biotin-labelled miR-30b-3p and pcDNA3.1-HNF1A-AS1-captured fraction (Fig. 6k, l). The level of HNF1A-AS1 was markedly decreased in the pull-down product isolated from the cells transfected with biotin-labelled miR-30b-3p and pcDNA3.1-PIK3CD 3′-UTR (Fig. 6m, n). Taken together, these results revealed that HNF1A-AS1 functioned as a ceRNA by competitively binding to miR-30b-3p to upregulate PIK3CD expression.

Moreover, expression of PIK3CD and miR-30b-3p was detected in mouse xenograft tumours. Overexpression of HNF1A-AS1 decreased the expression of miR-30b-3p, while promoting PIK3CD protein expression, confirming that HNF1A-AS1 functions as a ceRNA by competitively binding to miR-30b-3p to upregulate the expression of PIK3CD in vivo (Fig. 6o, p). Taken together, HNF1A-AS1 exhibited oncogenic properties by activating PI3K/AKT signalling through competitive binding to miR-30b-3p.

## Discussion

In this study, we showed that the expression of HNF1A-AS1 in patients with LNM was significantly higher than in patients without LNM. Moreover, ROC curve analysis revealed that HNF1A-AS1 expression could be used to distinguish between cases with LNM and those without LNM, and thus it could be used as a biomarker to predict LNM in GC. Moreover, HNF1A-AS1 enhanced migration and invasion abilities in vitro, which is consistent with previous reports,^12^ indicating that HNF1A-AS1 is a crucial oncogene in GC. Furthermore, we found that HNF1A-AS1 enhanced GC invasion, metastasis and angiogenesis in vivo. In addition, HNF1A-AS1 increased HUVEC and HLEC tube formation in vitro. It is known that angiogenesis promotes the initial development of primary malignant tumours, and is closely connected with infiltration and metastasis of cancer cells,^39^ and that new lymphatic vessels formed through lymphangiogenesis are responsible for cancer metastasis.^40^ Thus, we concluded that HNF1A-AS1 enhanced GC metastasis maybe due to its effect on angiogenesis and lymphangiogenesis.

To further explore the regulation mechanism of HNF1A-AS1 in GC metastasis, five genes, such as PIK3CD, PIK3R1, AKT1, AKT2 and AKT3, were chosen based on RT-qPCR and western blot results. HNF1A-AS1 increased the mRNA and protein expression of PIK3CD, AKT3 and PIK3CD, rather than AKT1 and AKT2, suggesting that HNF1A-AS1 exerts its oncogenic role by upregulating PIK3CD, PIK3R1 and AKT3. Moreover, HNF1A-AS1 increased the VEGF-A and VEGF-C secretion, and both are downstream of the PI3K/AKT signalling pathway.^26^ Furthermore, the PI3K inhibitor (NVP-BKM120) treatment rescued the tube formation of HUVECs and HLECs, and the migration abilities of GCs were enhanced by HNF1A-AS1, indicating that the HNF1A-AS1-induced biological functions might be mediated by PI3K/AKT signalling pathway. Many lncRNAs have recently been demonstrated to act as ceRNAs by competitively binding miRNA- responsive elements and impacting the expression levels of miRNA targets.^41^ For example, LncRNA PTAR competitively binds to miR-101-3p to regulate ZEB1 expression, and thus promote EMT and invasion–metastasis in serous ovarian cancer.^42^ LncRNA UICLM acts as a ceRNA by sponging miR-215 to regulate ZEB2 expression and promote colorectal cancer liver metastasis.^43^ RNA-FISH assays showed that HNF1A-AS1 was located in both the cytoplasm and nucleus. HNF1A-AS1 was hypothesised to function as a ceRNA by upregulating the expression of PIK3CD, PIK3R1 and AKT3. Bioinformatic analyses, luciferase assays, biotin-labelled miRNA pull-down assays and RIP assays were used to explore potential interactions between HNF1A-AS1 and miRNAs, and to confirm the direct binding capabilities of the predicted miRNA- responsive elements to the full-length HNF1A-AS1 transcript. As expected, miR-30b-3p was predicted to show complementary base pairing with HNF1A-AS1 and PIK3CD or AKT3. MiR-30b-3p significantly reduced the luciferase activity of the HNF1A-AS1 WT reporter vector. In addition, RIP assays indicated that endogenous HNF1A-AS1 pulldown with anti-ago2 was specifically enriched compared with the IgG antibody. RNA-FISH showed that HNF1A-AS1 and miR-30b-3p were co-localised in the cytoplasm. MiR-30b-3p pull-down assays suggested more than a tenfold enrichment of HNF1A-AS1 in the biotin-labelled miR-30b-3p-captured fraction compared with the negative control. Furthermore, miR-30b-3p abolished the HNF1A-AS1-mediated biological effects. Similarly, PIK3CD was revealed to be a target gene of miR-30b-3p. Taken together, these data indicated that HNF1A-AS1 can serve as a ceRNA to sequester miR-30b-3p, thereby protecting the target gene, PIK3CD, from repression, and ultimately promoting tumorigenesis and progression in GC.

In summary, lncRNA-HNF1A-AS1 acts as a ceRNA that activates PI3K/AKT signalling by competitively binding to miR-30b-3p and exhibits oncogenic properties in GC (Fig. 6q). Understanding the role of HNF1A-AS1 in GC could provide a potential therapeutic target for treating GC.

## Supplementary information

supplenmentary Data

## Data Availability

All data generated or analysed during this study are included in this published article and its supplementary information files.
