# Supplementary material for: LncRNA-HNF1A-AS1 functions as a competing endogenous RNA to activate PI3K/AKT signalling pathway by sponging miR-30b-3p in gastric cancer
Source: Br J Cancer. 2020 Apr 27;122(12):1825–36. doi: 10.1038/s41416-020-0836-4 (PMC7283217; doi:10.1038/s41416-020-0836-4)
Supplement: Supplementary file 1 — supplenmentary Data [file 41416_2020_836_MOESM1_ESM.docx]

**Supplementary Figure**


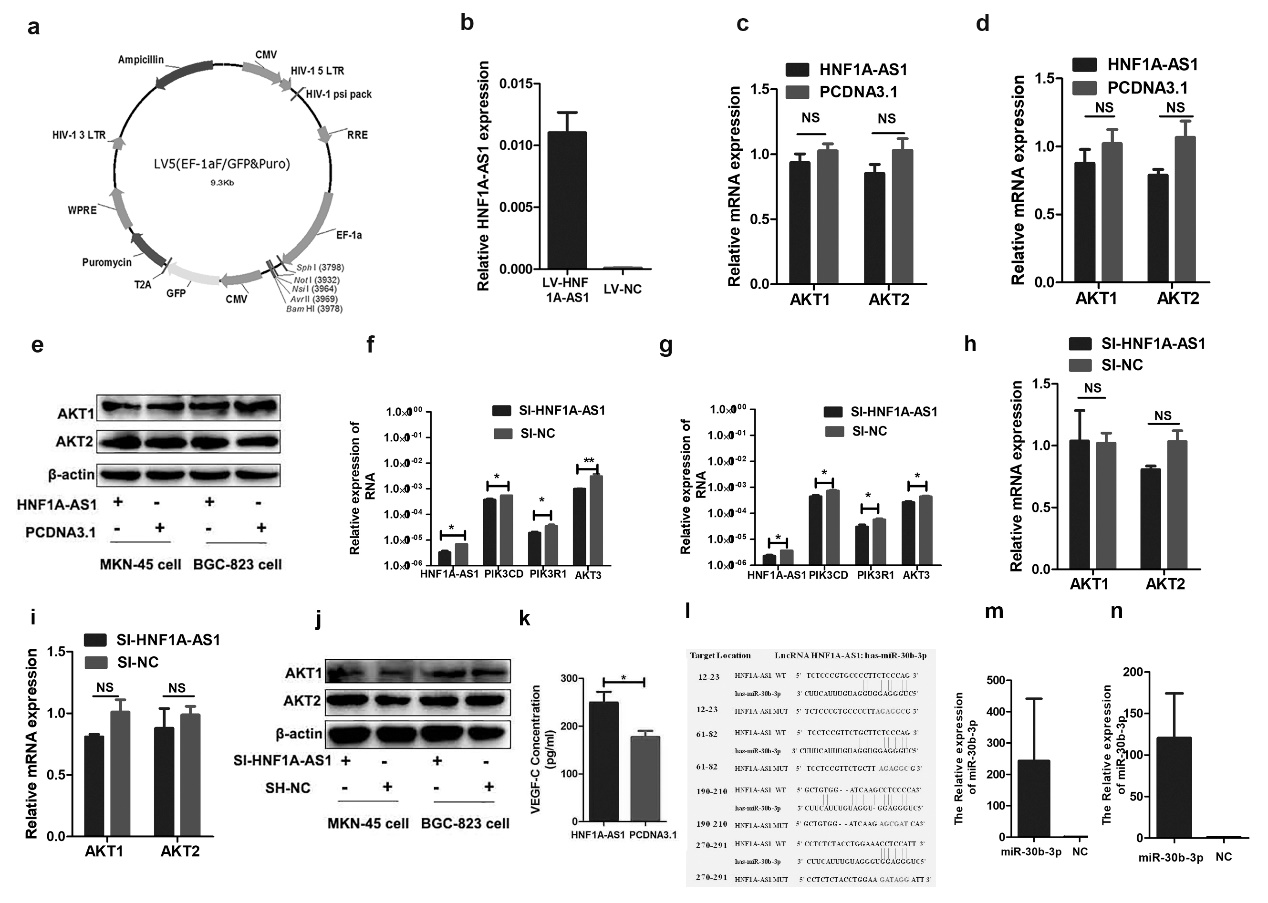


**Figure legend:**

**Supplementary Fig. 1 HNF1A-AS1 functions as a ceRNA with miR-30b-3p.**

**a** The full Sequence Map for LV5.

**b** HNF1A-AS1 was overexpressed stably *in vivo* by RT-qPCR.

**c-d** RT-qPCR assays showed that HNF1A-AS1 overexpression did not increased AKT1 and AKT2 mRNA level in MKN-45 cells (c) and BGC-823 cells (d).

**e** Western blotting assays demonstrated that HNF1A-AS1 overexpression did not enhanced AKT1 and AKT2 protein expression.

**f-g** RT-qPCR assays demonstrated that knockdown of HNF1A-AS1by si-RNA decreased PIK3CD, PIK3R1 and AKT3 mRNA expression in MKN-45 cells (f) and BGC-823 cells (g).

**h-i** RT-qPCR assays indicated that knockdown of HNF1A-AS1by si-RNA did not decreased AKT1 and AKT2 mRNA expression in MKN-45 cells (h) and BGC-823 cells (i).

**j** Western blotting assays demonstrated that knockdown of HNF1A-AS1by si-RNA did not decreased AKT1 and AKT2 protein expression.

**k** The secretion level of VEGF-C significantly enhanced in the culture supernatant from the HNF1A-AS1-transfected BGC-823 cells, compared to the negative control groups, detected by ELISA.

**l** Predicted and mutant binding sites of miR-30b-3p on HNF1A-AS1. The red nucleotides are the mutant seed sequence of miR-30b-3p.

**m-n** MiR-30b-3p was successfully overexpressed in MKN-45 (m) and BGC-823 cells (n).

*P < 0.05, ** P < 0.01, *** P < 0.001
